# The effect of background music on children's scientific creativity

**DOI:** 10.3389/fpsyg.2026.1773321

**Published:** 2026-05-20

**Authors:** Jiankun Liu

**Affiliations:** School of Music, Shangqiu Normal University, Shangqiu, Henan, China

**Keywords:** background music, children, scientific creativity, music tempo, timing

## Abstract

Previous studies on background music and children's creativity have yielded conflicting conclusions, which may be due to the modulation of the effects of background music by task nature, music attributes, broadcast timing, and individual differences. We explored the effects of background music tempo and broadcast timing on prototype-based inventive thinking through two experiments. The results show that slow tempo background music enhances the children's retention of prototype knowledge, while fast tempo background music during the inventive thinking process promotes the fluency and flexibility of scientific inventive thinking but hinders novelty. Playing slow tempo background music during the learning of scientific knowledge can enhance the retention of scientific knowledge and improve scientific inventive thinking. Playing fast tempo background music during the inventive thinking process can change the attention pattern, processing efficiency, and information processing mode, thereby improving the fluency and flexibility of scientific inventive thinking.

## Introduction

Scientific creativity refers to the ability to generate novel ideas or products that are relevant to the environment, possess scientific value, or hold significance. It serves as a crucial factor for a country's survival and success in an evolving and competitive global landscape (Hu and Yu, 2002). With the rapid development of the Internet and electronic devices, accessing music resources has become increasingly convenient for children's. Concurrently, it has become more common for people to engage in work or study while listening to background music, which played when one is focused on tasks unrelated to the music itself (Lehmann and Seufert, 2017). As an environmental stimulus, background music can influence learning performance and cognitive tasks by impacting children's emotional state and level of attention (de la Mora Velasco and Hirumi, 2020).

Regarding its effect on divergent thinking tasks, previous studies predominantly employed multifaceted objectives and consistently concluded that playing background music enhances individual performance both prior to and during task execution (Hosseini et al., 2019; Ritter and Ferguson, 2017; Xia et al., 2023). However, there have been inconsistent findings regarding the impact of background music on convergent thinking tasks. Some studies suggest that playing background music does not significantly improve children's performance in aggregated thinking tasks before or during task completion (Ilie and Thompson, 2011). Conversely, other studies indicate that playing background music during task completion diminishes children's performance in long-distance association tasks (Threadgold et al., 2019).

The relationship between background music and creativity is not only influenced by the nature of the creative task but also closely tied to specific properties of the background music, such as its rhythm and duration. Consequently, there are two potential explanations for the inconsistent findings. Firstly, from a cognitive processing perspective, some researchers have employed the cognitive resource model and irrelevant sound effects to elucidate that background music diminishes children's cognitive processing capacity (Doyle and Furnham, 2012; Tillmann, 2007). As individual cognitive resources are limited, having accompanying background music during a task depletes these resources and increases cognitive load (Experiment 2 Hypothesis 2). If an individual's processing demands exceed their available cognitive resources, it leads to interference with their cognition performance (Kahneman, 2000; Rey, 2012). Additionally, slow-tempo music requires more time to convey the same amount of information compared to fast-tempo music. This results in children needing to allocate additional cognitive resources (Experiment 1 hypothesis 2). From this perspective, the presence of background music hinders learners' cognitive processing and consequently impacts their performance in creative cognitive tasks. Conversely, considering emotional arousal, the awakening-mood hypothesis proposed by Thompson et al. (2001) suggests that music can enhance children's arousal and mood, thereby improving their cognitive performance. The rhythm of the music plays a regulatory role, with fast tempo music having a greater impact on children's arousal compared to slow tempo music (Experiment 1 Hypothesis 1). This theoretical proposition has been substantiated in various tasks such as reading, memory, and language where it has been observed that fast-paced music facilitates faster completion of general cognitive tasks compared to slower paced music (Lange et al., 2018). Consequently, from this standpoint, the inclusion of background music supports learners' cognitive processing and subsequently influences their performance in creative cognitive tasks (Experiment 1 Hypothesis 3).

The above two theories and related empirical research findings present contradictory results. Is there a specific form of background music that can effectively balance learners' cognitive resources, while also enhancing arousal, mood, and ultimately improving cognitive performance? Therefore, the timing of background music becomes a crucial variable in this context. According to the Mozart effect theory proposed by Jenkins (2001), listening to Mozart's music prior to engaging in a cognitive task can evoke positive emotions in children and consequently enhance cognitive processing (Experiment 2 Hypothesis 1). Hence, when background music is played before initiating a creative task, it not only induces positive emotions and facilitates subsequent cognitive tasks but also prevents potential overload on cognitive resources caused by the presence of background music (Experiment 2 Hypothesis 3).

In previous studies, the creative tasks examined were limited to domains with poor knowledge, thereby neglecting the influence of knowledge on invention and creation. Researchers have long acknowledged that knowledge experience forms the foundation of creativity. For instance, Gajda et al.'s (2017) meta-analysis revealed a positive correlation between student academic performance and creativity (*r* = 0.22). The task of scientific invention and creation, known as the archetypal heuristic paradigm, falls within the domain rich in knowledge (Zhu et al., 2012). Within this paradigm, learners are required to acquire relevant knowledge including potential archetypes before encountering new problem scenarios. By drawing analogies between new problems and previously encountered ones through searching for similar archetypes from past experiences, innovative solutions can be proposed (Yang W. J. et al., 2022). Henceforth, this study employs a prototyping-inspired creation task to investigate how background music rhythm and playing time impact both knowledge retention and invention creation.

## Experiment 1

### Purpose

Experiment 1 was designed to investigate the effect of background music rhythm on the creation of inventions.

### Methods

#### Subjects and experimental design

A total of 60 children (32 males and 28 females) aged 8 to 12 years old (*M* = 10.40, *SD* = 1.2 years old) were randomly recruited from a primary school. Written informed consent was obtained from parents or guardians prior to participation. Subject design, independent variable is background music, including three levels: no background music, slow rhythm background music, and fast rhythm background music.

#### Background and music of video learning materials

Nine topics (Sun, 2012) were randomly selected from the Material Bank of Creative and Invention Experiment Problems. According to the scientific knowledge involved in the topics, three teaching videos were made. Each video taught three knowledge points, and the length of each video was about 11 min. The theme music from Spirited Away, composed by Joe Hisaishi, was used as the background music. The original rhythm of the music is 96 bpm. After the variable speed treatment, the slow tempo is 76 bpm and the fast tempo is 119 bpm (Bramley et al., 2016).

### Tools

#### Emotional valence and arousal questionnaire

Emotional grid questionnaire was used to measure titer and arousal (Russell et al., 1989), with 1 representing the lowest valence/arousal and 9 representing the highest titer/arousal. In this study, the difference between before and after the experiment was used to investigate the influence of background music on emotional titer and arousal.

#### Knowledge retention test

The self-designed learning performance test measures subjects' understanding of the learning content under three experimental conditions, including 45 items. In this study, the Cronbach's α coefficient of this questionnaire was 0.71.

#### Cognitive load test

Students' cognitive load was assessed using the Learning State scale compiled by Stull et al. (2018), which was scored at 7 points. The question “Please rate how much effort you put into understanding the material” assesses students' cognitive load.

The subjects' scientific creativity was assessed in terms of fluency, flexibility, and novelty through the creation of teaching videos, using a set of 9 questions. Fluency scores were based on the number of valid ideas generated by each subject. Flexibility scores were calculated based on the range of domain categories covered by their views. Novelty scores were determined by the proportion of their ideas among all participants' ideas. Ideas appearing in less than 5% received 3 points, those appearing between 5% and 10% received 2 points, and those appearing in more than 10% received 1 point (Hu and Adey, 2002). The reliability coefficients for fluency, flexibility, and consistency ratings conducted by two trained raters were 0.86, 0.89, and 0.90, respectively.

### Procedure

The experimental procedure comprised three phases and lasted approximately 2 h. Prior to commencing the formal experiment, participants provided informed consent and completed demographic questionnaires. Firstly, they were instructed to fill out the Emotion Grid questionnaire. Secondly, subjects watched an 11-min lesson under three different experimental conditions (study video), with the presentation order randomized. Finally, after each instructional video, participants completed emotional grid questionnaires, knowledge retention tests, cognitive load questionnaires, and creative tasks.

### Results

Repeated measures analysis of variance was employed to examine the impact of background music rhythm on scientific and creative activities. Descriptive statistics are presented in Table 1 while ANOVA results are displayed in Table 2.

**Table 1:** Descriptive statistical results of Experiment 1.

Dependent variable	No background music		Slow background music		Fast background music	
	*M*	*SD*	*M*	*SD*	*M*	*SD*
Emotion						
Valence	5.68	1.72	6.31	1.44	6.33	1.53
Arousal	3.80	1.67	4.05	1.85	3.85	1.92
Cognitive load	3.73	0.18	3.58	0.20	3.87	0.23
Cognitive retention	18.90	4.39	28.75	4.32	26.13	3.90
Scientific creativity						
Fluency	3.24	0.48	2.98	0.30	3.30	0.62
Flexibility	3.05	0.36	2.96	0.29	3.13	0.39
Novelty	5.58	0.89	5.97	1.10	6.59	1.32

**Table 2:** Analysis of variance results of Experiment 1.

Dependent variable	*SS*	*df*	*MS*	*F*	*p*
Emotion					
Valence	3.74	2	1.87	2.073	>0.05
Arousal	2.63	2	1.32	1.586	>0.05
Cognitive load	3.06	2	1.53	4.39	< 0.05
Knowledge retention	2,525.83	2	1,262.92	63.28	< 0.001
Scientific creativity					
Fluency	3.01	2	1.51	6.458	< 0.01
Flexibility	0.81	2	0.41	4.010	< 0.05
Novelty	29.23	2	14.62	15.67	< 0.001

#### Emotional valence and arousal degree

Under different experimental conditions, the subjects did not show significant differences in both the emotional valence and arousal dimensions, *F*_(2, 118)_ = 2.07, *p* > 0.05, *F*_(2, 118)_ = 1.59, *p* > 0.05.

#### Cognitive load

There were significant differences in cognitive load under different experimental conditions, *F*_(2, 118)_ = 4.39, *p*<*0.05*, η^2^ = 0.07. The results of Bonferroni *post hoc* analysis indicated (similarly), that the cognitive load under the slow-paced background music condition was significantly lower than that under the no-background music and fast-paced background music conditions (*MD* = −0.26, *p*<*0.05, MD* = −0.29, *p*<*0.05*).

#### Knowledge retention

There were significant differences under different experimental conditions, *F*_(2, 118)_ = 63.28, *p*<*0.001*, η^2^ = 0.61. *Post hoc* test analysis revealed that knowledge retention under the slow-paced background music condition was significantly better than that the fast-paced background music condition and the no-background-music condition (*MD* = 2.58, *p*<*0.001, MD* = 8.92, *p*<*0.001*), and the fast-paced background music condition was significantly better than the no-background-music condition (*MD* = 6.33, *p*<*0.001*).

#### Scientific creativity

Due to the heterogeneity of the variance of fluency (*p* < 0.05), the analysis results after Greenhouse-Geisser correction indicated that there were significant differences under different experimental conditions, *F*_(2, 106)_ = 6.31, *p* = 0.001, η^2^ = 0.11. The fluency score under the fast-paced background music condition was significantly higher than that under the slow-paced background music condition (*MD* = 0.32, *p* = 0.001), and the fluency score under the no-background music condition was significantly higher than that under the slow-paced background music condition (*MD* = 0.17, *p* = 0.051). There were no significant differences among other experimental conditions (*ps* > 0.05).

The results of flexible variance analysis indicate that there are under different experimental conditions. There was a significant difference, *F*_(2, 118)_ = 4.04, *p*<*0.05*, η^2^ = 0.06. The flexibility score under the fast-paced background music condition was marginally significantly higher than that under the slow-paced background music condition (*MD* = 0.15, *p* = *0.056*), and there were no significant differences among other experimental conditions (*ps* > 0.05).

The results of novelty variance analysis indicated that there were significant differences under different experimental conditions. The novelty scores differed significantly across conditions, *F*_(2, 118)_ = 15.67, *p*<*0.001*, η^2^ = 0.21. *Post hoc* comparisons indicated that novelty scores under the fast-tempo condition were significantly higher than those under the no-music condition (*MD* = 0.98, *p*<*0*.001). The difference between the slow-tempo and no-music conditions was not statistically significant (*p* > 0.05).

#### Correlation analysis

The results of correlation analysis of variables under different experimental conditions are shown in Tables 3–5. Given the exploratory nature of these correlation analyses and the number of comparisons conducted, the results should be interpreted with caution.

**Table 3:** Correlation analysis of each variable under the condition without background music.

Variable	Emotional valence	Emotional arousal	Knowledge retention	Cognitive load	Fluency	Flexibility
Emotional valence						
Emotional arousal	0.13					
Cognitive load	−0.003	−0.005				
Knowledge retention	−0.03	−0.11	−0.02			
Fluency	−0.18	−0.15	0.14	0.10		
Flexibility	−0.21	< 0.01	< 0.01	0.24	0.54^**^	
Novelty	−0.18	−0.27^*^	−0.07	0.27^**^	0.37^**^	0.47^**^

^*^p < 0.05, ^**^p < 0.01.

**Table 4:** Correlation analysis of each variable under the condition slow background music.

Variable	Emotional valence	Emotional arousal	Knowledge retention	Cognitive load	Fluency	Flexibility
Emotional valence						
Emotional arousal	0.27^*^					
Cognitive load	−0.14	−0.14				
Knowledge retention	0.21	−0.05	−0.10			
Fluency	0.21	0.02	0.04	0.04		
Flexibility	0.21	0.02	0.04	0.04	0.84^**^	
Novelty	0.11	−0.27^*^	0.01	0.22	0.37^**^	0.47^**^

^*^p < 0.05, ^**^p < 0.01.

**Table 5:** Correlation analysis of each variable under the condition fast background music.

Variable	Emotional valence	Emotional arousal	Knowledge retention	Cognitive load	Fluency	Flexibility
Emotional valence						
Emotional arousal	−0.15					
Cognitive load	−0.09	−0.001				
Knowledge retention	0.02	−0.28^*^	−0.09			
Fluency	−0.27^*^	0.17	0.06	0.18		
Flexibility	−0.09	−0.02	0.03	0.27^*^	0.68^**^	
Novelty	−0.15	−0.002	0.11	0.29^*^	0.76^**^	0.55^**^

^*^p < 0.05, ^**^p < 0.01.

### Summary and discussion of the results of experiment

The background music of different rhythms did not induce differences in the valence and arousal of children's emotions. The cognitive load under the slow-rhythm background music condition was the lowest and lower than that the no-background-music condition. The knowledge retention effect under the slow-rhythm background music condition was the best, followed by that under the fast-rhythm background music condition. In terms of creativity and invention, the fluency under the fast-rhythm background music condition was the best, followed by that under the no-background-music condition. The flexibility and novelty under the fast-rhythm background music condition were higher than those under the slow-rhythm background music. Under the fast-rhythm background music condition, there was a positive correlation between the retention of scientific knowledge and the flexibility and novelty of creativity and invention.

Although different types background music do not induce different emotional valences and arousal levels, they do affect an individual's cognitive load, the retention effect of scientific knowledge, and thereby influence creativity and invention. Background music may not improve the retention effect of scientific knowledge and technological creation and invention through emotional arousal (Doyle and Furnham, 2012; Toplyn and Maguire, 1991). Experiment 1 also found that the mechanism of action of background music with different rhythms varies. Under slow-paced music, children have a lower cognitive load and can invest more cognitive resources in learning materials, thereby improving the level of knowledge retention. At the same time, the retention effect of scientific knowledge with background music (regardless of rhythm speed) is better than that without background music, indicating that background music can indeed promote cognitive performance. Finally, the retention of scientific knowledge and creativity and invention under fast-paced background music are positively correlated, and the flexibility and novelty are higher than those under slow-paced background music. This may be because fast-paced background music can change the attention mode, processing speed, and processing method of children in the task of creativity and invention (Lange et al., 2018; Zabelina et al., 2015), thereby improving flexibility and novelty.

## Experiment 2

### Purpose

Since Experiment 1 found that fast-paced background music was more conducive to children's creative inventions, Experiment 2 aimed to explore the influence of the playing timing of fast-paced background music on scientific creative activities.

### Methods

#### Subjects and experimental design

Ninety children (42 male) were randomly recruited from a primary school in Henan Province. There were 48 girls (aged 8 to 12 years old, *M* = 10.40 years old, *SD* = 1.31 years old). Participants were randomly assigned to one of three conditions (*n* = 30 per group). Their parents signed the informed consent form and received a compensation of 30 yuan. This experiment adopted a between-subjects design. The independent variable was the playing time, including three levels: playing music before video learning, playing music during video learning, and playing music during creative invention. The instrument has been previously used in studies of scientific creativity and demonstrates acceptable construct validity (Sun, 2012).

#### Background and music of video learning materials

The same as Experiment 1.

### Tools

The same as Experiment 1.

### Procedure

The experiment will take approximately 1 h. The children fill in the demographic information, the Scientific Creativity Test and the Emotional Grid Questionnaire, the children are randomly assigned to the music-playing group before video learning, the music-playing group during video learning, and the music-playing group during creative invention. In the music-playing group before video learning, they will first listen to the background music for 30 min, then watch the teaching video for about 11 min, and finally fill out the Emotional Grid Questionnaire, the Knowledge Retention Test, the Cognitive Load Questionnaire and the Creative Invention Task. In the music-playing group during video learning, the background music is played during the video viewing process, and then they fill out the Emotional Grid Questionnaire, the Knowledge Retention Test, the Cognitive Load Questionnaire, and the Creative Invention Task. In the music-playing group during creative invention, they complete the creative invention task with the background music on.

### Results

Analysis of variance was used to investigate the influence of the playing time of background music on creative inventions. The descriptive statistics and the results of analysis of variance are presented in Tables 6, 7, respectively.

**Table 6:** Descriptive statistical results of Experiment 2.

Dependent variable	Playing before video study		Playing during video study		Playing after video study	
	***M***	***SD***	***M***	***SD***	***M***	***SD***
Emotion						
Valence	5.80	1.88	6.33	1.54	6.90	1.20
Arousal	4.57	1.83	3.85	1.92	3.88	1.98
Cognitive load	4.18	0.85	3.87	0.95	5.07	0.83
Cognitive retention	23.87	4.05	26.13	3.91	23.40	4.15
Scientific creativity						
Fluency	3.40	0.68	3.38	0.60	4.30	1.62
Flexibility	3.15	0.46	3.13	0.39	3.73	1.39
Novelty	7.58	1.89	6.53	1.32	6.09	1.42

**Table 7:** Analysis of variance results of Experiment 2.

Dependent variable	*SS*	*df*	*MS*	*F*	*p*
Emotion					
Valence	17.10	2	8.85	3.554	< 0.05
Arousal	11.38	2	5.69	1.508	>0.05
Cognitive load	34.71	2	17.35	20.353	< 0.001
Knowledge retention	157.90	2	78.95	4.930	< 0.01
Scientific creativity					
Fluency	11.23	2	5.61	7.403	0.01
Flexibility	8.73	2	4.37	8.176	< 0.01
Novelty	16.23	2	8.11	3.702	< 0.05

#### Emotional valence and arousal degree

There were significant differences under different experimental conditions, *F*_(2, 87)_ = 3.55, *p*<*0.05*. The emotional valence of learners in the background music playing group during creation and invention was significantly higher than that in the background music playing group before video learning (*MD* = 1.07, *p*<*0.05*). There was no significant difference in emotional arousal under different experimental conditions, *F*_(2, 87)_ = 1.51, *p* > 0.05.

#### Cognitive load

There were significant differences under different experimental conditions, *F*_(2, 87)_ = 20.35, *p*<*0.001*, η^2^ = 0.26. The cognitive load of learners in the background music-playing group in creative invention was significantly higher than that in the background music-playing group in video learning and the background music-playing group before video learning (*MD* = 1.31, *p*<*0.001*; *MD* = 0.99, *p*<*0.001*).

#### Knowledge retention

There were significant differences under different experimental conditions, *F*_(2, 87)_ = 4.93, *p* < 0.01, η^2^ = 0.08. The knowledge retention of learners in the background music-playing group in creative invention was significantly lower than that of the background music-playing group in video learning and the background music-playing group before video learning (*MD* = −2.70, *p* < 0.01; *MD* = −2.53, *p* > 0.05).

#### Scientific creativity

There were significant differences under different experimental conditions, *F*_(2, 87)_ = 7.40, *p* = 0.001, η^2^ = 0.11. The fluency of the background music group in the invention was significantly higher than that of the background music group in video learning and the background music group before video learning (*MD* = 0.73, *p*<*0.001*; *MD* = 0.63, *p* < 0.01).

There were significant differences under different experimental conditions, *F*_(2, 87)_ = 8.18, *p*<*0.001*, η^2^ = 0.12. The flexibility of the background music-playing group in creative invention was significantly higher than that of the background music-playing group in video learning and the background music-playing group before video learning (*MD* = 0.63, *p* = 0.001; *MD* = 0.60, *p* < 0.01).

There were significant differences under different experimental conditions, *F*_(2, 87)_ = 3.69, *p*<*0.05*, η^2^ = 0.06. The novelty of the background music-playing group before video learning was significantly higher than that of the background music-playing group in the creative invention (*MD* = 1.03, *p*<*0.05*).

#### Correlation analysis

The results of correlation analysis of variables under different experimental conditions are shown in Tables 8–10.

**Table 8:** Correlation analysis of each variable under the playing conditions before video learning.

Variable	Emotional valence	Emotional arousal	Knowledge retention	Cognitive load	Fluency	Flexibility
Emotional valence						
Emotional arousal	−0.07					
Cognitive load	0.36	0.04				
Knowledge retention	0.15	−0.20	−0.04			
Fluency	0.20	−0.06	−0.08	0.29		
Flexibility	0.20	−0.24	−0.19	0.30	0.78^**^	
Novelty	0.31	−0.06	0.07	0.26	0.90^**^	0.73^**^

^*^p < 0.05, ^**^p < 0.01.

**Table 9:** Correlation analysis of each variable under the playing conditions during video learning.

Variable	Emotional valence	Emotional arousal	Knowledge retention	Cognitive load	Fluency	Flexibility
Emotional valence						
Emotional arousal	−0.06					
Cognitive load	0.15	0.09				
Knowledge retention	−0.03	−0.23	−0.08			
Fluency	−0.04	−0.12	0.06	0.18		
Flexibility	0.02	−0.29^*^	0.03	0.27^*^	0.68^**^	
Novelty	−0.05	0.02	0.11	0.29^*^	0.76^**^	0.55^**^

^*^p < 0.05, ^**^p < 0.01.

**Table 10:** Correlation analysis of each variable under the playing conditions after video learning.

Variable	Emotional valence	Emotional arousal	Knowledge retention	Cognitive load	Fluency	Flexibility
Emotional valence						
Emotional arousal	−0.29					
Cognitive load	0.41^*^	−0.11				
Knowledge retention	0.12	0.07	0.12			
Fluency	0.24	−0.10	0.01	0.35		
Flexibility	0.21	−0.22	−0.04	0.22	0.94^**^	
Novelty	0.15	−0.12	−0.13	0.38^*^	0.87^**^	0.88^**^

^*^p < 0.05, ^**^p < 0.01.

### Summary and discussion of Experiment 2 results

The results of Experiment 2 indicate that, compared with the group with background music played before and during video learning, the group with background music played during creative invention has the highest cognitive load, the poorest knowledge retention effect, and the highest fluency and flexibility in creative invention. In addition, the emotional effect of the group with background music played during creative invention is higher than that of the group with background music played before video learning, but the novelty of creative invention is lower. The research results also found that when fast-paced background music is played during video learning, the retention effect of scientific knowledge is positively correlated with technological creative invention. This indicates that although fast-paced background music increases cognitive load, affects cognitive processing, and undermines the novelty of scientific creative invention, it can improve the fluency and flexibility of scientific creative invention by changing the way of attention mode, processing speed, and information processing (Lange et al., 2018; Zabelina et al., 2015).

## Discussion

Experiment 1 and Experiment 2 did not verify the arousal-mood hypothesis and the Mozart Effect. The speed of the background music rhythm and the playing timing did not induce different emotional valences and arousal levels. Although some studies recognized background music can have an impact on an children's emotional valence and arousal level, thereby influencing their cognitive performance and creative thinking (Ilie and Thompson, 2011; Yao et al., 2018; Ritter and Ferguson, 2017). However, some studies suggested that the relationship between music and emotional arousal was complex, and the emotions that music intends to convey are not consistent with those experienced by the listeners (Ma et al., 2013; Yang J. M. et al., 2022; Zhang et al., 2022). Some empirical studies have also found that background music does not improve general cognitive performance or even creative thinking performance through emotional arousal (Doyle and Furnham, 2012; Toplyn and Maguire, 1991).

Playing music while watching videos had the best knowledge retention effect. This might be because playing slow-paced background music can reduce cognitive load, which is inconsistent with the cognitive resource model and the irrelevant sound effect (Kahneman, 2000; Rey, 2012), but to some extent supports the Mozart Effect (Jenkins, 2001), that is, background music can make children feel cheerful, pleasant, etc., thereby reducing the difficulty of their perception tasks and the degree of psychological effort, and improving the effect of knowledge retention. As for the playing timing, the knowledge retention effect of the background music playing group in video learning and the background music playing group before video learning was better than that of the background music playing group in creative invention. This once again suggests the promoting effect of playing background music on knowledge learning.

Regarding creativity and invention, children who play fast-paced background music exhibit the highest levels of fluency, flexibility, and novelty. This may be due to the fact that fast-paced background music alters an individual's attentional patterns and information processing methods. According to the broad attention span hypothesis of creativity (Zabelina et al., 2015), background sounds reduce the total amount of attention children use for problem-solving tasks, leading to a state of attentional diffusion, which is more conducive to the resolution of creative problems (Zabelina et al., 2016). Additionally, previous studies have consistently found that fast-paced background music can improve an individual's cognitive processing speed and efficiency (Kämpfe et al., 2011; Lange et al., 2018). Fast-paced background music can accelerate the processing speed of children in creative invention tasks, thereby enhancing their fluency and flexibility, and ultimately improving their novelty. Therefore, compared to no background music and slow-paced background music, the flexibility and novelty are highest under the condition of fast-paced background music.

Regarding the playing timing, the background music group in the creation and invention task had significantly higher fluency and flexibility than the background music group in the video learning task and the background music group before video learning, but the novelty was lower than the other two groups. This might be because when completing the invention and creation tasks, fast-paced music can accelerate the processing speed of children. Although this can improve the fluency and flexibility of children when completing the invention and creation tasks, due to the large amount of information presented within the unit time of the fast-paced background music, the cognitive load increases, thereby reducing cognitive inhibition. During the process of generating creative ideas, children generate novel and unique viewpoints by establishing connections between related but weakly associated concepts (Mednick, 1962). Under high cognitive load, the reduction of children's cognitive inhibition function on the one hand increases the speed of generating creative ideas, but on the other hand, it weakens the inhibition of meaningless ideas, which is not conducive to the generation of novel ideas (Benedek et al., 2014). Additionally, although existing studies have shown that playing music before divergent thinking tasks can improve their performance, most of these divergent thinking tasks are completed within a short period of time, so the continuous effect of background music remains within the validity period. In this study, the prototype heuristic paradigm was used, requiring children to complete scientific creative solution tasks. In this task, children need to use divergent thinking to consider various possible solutions during the idea generation stage, and then need to use convergent thinking to evaluate and integrate the solutions, involving the collaborative work of divergent thinking and convergent thinking (de Vries and Lubart, 2019). Therefore, compared with divergent thinking tasks, scientific creation and invention tasks require longer cognitive processing time. Longer processing time means that the effect of playing background music before the task attenuates over time and may even be insufficient to sustain until the completion of the creative task, which might be the reason for the lower fluency and flexibility in the background music group before learning.

Regarding the role of knowledge in creation and invention. Firstly, through correlation analysis, it was found that the better the knowledge retention effect of an individual is, the higher their flexibility and novelty are. Secondly, playing slow-paced background music when watching instructional videos and playing fast-paced background music before creation and invention is more conducive to knowledge retention, indicating that playing background music has a promoting effect on knowledge retention and is conducive to creation and invention. Fast-tempo music was associated with higher fluency and novelty. This result is consistent with previous research findings. Scientific knowledge is the prerequisite and foundation of creation and invention, and an indispensable “raw material” in the process of creation and invention. Creation and invention are the recombination of existing scientific knowledge to form novel knowledge connections (Hu and Adey, 2002). In creative activities in special fields, children with more knowledge experience are more likely to conduct distant associations through extensive cognitive retrieval and increased sensitivity to external cues, eventually generating highly creative products (Gilhooly et al., 2015). In the prototype heuristic paradigm, children need to first understand and represent new problems, then search and match existing prototype paradigms in their memory, and finally achieve creative problem-solving through transfer (Yang W. J. et al., 2022). These processes clearly require better knowledge retention. This also indicates that adopting knowledge-rich problems and exploring the impact of knowledge retention on scientific creation is more in line with the real scenarios of scientific problem-solving and is conducive to improving the ecological validity of research.

## Limitations and future directions

While the present study provides meaningful insights into the relationship between background music and children's scientific creativity, several limitations should be acknowledged. First, although the sample size met statistical requirements, it was relatively modest and drawn from a single educational context, which may limit the generalizability of the findings. Second, only musical tempo was manipulated in this study, whereas other musical parameters such as volume, timbre, and harmonic complexity were not controlled. These factors may also influence cognitive processing and creative performance. Third, individual differences, including music preference, baseline attention levels, and emotional sensitivity, were not examined and may have contributed to variability in responses. Future research could adopt larger and more diverse samples, incorporate additional musical variables, and explore underlying cognitive and affective mechanisms using multi-method approaches.

## Data Availability

The original contributions presented in the study are included in the article/supplementary material, further inquiries can be directed to the corresponding author.
